# Caught between pity, explicit bias, and discrimination: a qualitative study on the impact of stigma on the quality of life of persons living with sickle cell disease in three African countries

**DOI:** 10.1007/s11136-023-03533-8

**Published:** 2023-10-27

**Authors:** Nchangwi Syntia Munung, Marsha Treadwell, Karen Kengne Kamga, Jemima Dennis-Antwi, Kofi Anie, Daima Bukini, Julie Makani, Ambroise Wonkam

**Affiliations:** 1 Division of Human Genetics, Faculty of Health Sciences, University of Cape Town, Cape Town, South Africa; 2 Department of Pediatrics/Division of Hematology, University of California San Francisco, Oakland, CA USA; 3 University of Yaoundé 1, Yaoundé, Cameroon; 4 Sickle Cell Foundation of Ghana, Accra, Ghana; 5 London Northwest University Healthcare (NHS) Trust, Harrow, UK; 6 Imperial College London, London, UK; 7 Muhimbili University of Health and Allied Sciences, Dar Es Salaam, Tanzania; 8 McKusick-Nathans Institute and Department of Genetic Medicine, Johns Hopkins University School of Medicine, Baltimore, USA

**Keywords:** Sickle cell disease, Stigma, Discrimination, Explicit bias, Pity, Africa, Quality of life, Ghana, Cameroon, Tanzania

## Abstract

**Purpose:**

Sickle cell disease (SCD) is an inherited blood disorder characterized by unpredictable episodes of acute pain and numerous health complications. Individuals with SCD often face stigma from the public, including perceptions that they are lazy or weak tending to exaggerate their pain crisis, which can profoundly impact their quality of life (QoL).

**Methods:**

In a qualitative phenomenological study conducted in Cameroon, Ghana, and Tanzania, we explored stakeholders’ perceptions of SCD-related stigma using three analytical frameworks: *Bronfenbrenner’s Ecological Systems Theory*; *The Health Stigma and Discriminatory Framework;* and *A Public Health Framework for Reducing Stigma.*

**Results:**

The study reveals that SCD-related stigma is marked by prejudice, negative labelling and social discrimination, with derogatory terms such as sickler, *ogbanje* (one who comes and goes), *sika besa* (money will finish), *ene mewu* (I can die today, I can die tomorrow), *vampire* (one who consumes human blood), and Efiewura (landlord-*of the hospital*), commonly used to refer to individuals living with SCD. Drivers of stigma include frequent crises and hospitalizations, distinct physical features of individuals living with SCD, cultural misconceptions about SCD and its association with early mortality. Proposed strategies for mitigating stigma include public health education campaigns about SCD, integrating SCD into school curricula, healthcare worker training and community engagement.

**Conclusion:**

The results highlight the importance of challenging stigmatizing narratives on SCD and recognizing that stigmatization represents a social injustice that significantly diminishes the QoL of individuals living with SCD.

## Introduction

Sickle cell disease (SCD) is the most prevalent hereditary blood disease in humans. It is caused by a genetic mutation in the beta-globin gene [1]. This gene encodes a component of haemoglobin (Hb), the protein in red blood cells (RBCs) responsible for transporting oxygen to the body’s organs. The mutation causes RBCs to become deformed and rigid, taking on a sickle or banana shape. They deformed RBCs tend to block small blood vessels causing pain and damage to various organs. These abnormal RBCs also have a much shorter lifespan (about 20 days instead of 120 days) and are prone to rapid breakdown, leading to anaemia.

Globally, approximately 25 million people live with SCD, the majority residing in Africa [2, 3]. Africa also accounts for about 78% of the 515,000 yearly SCD births worldwide [4], and the under-five mortality in Africa due to SCD ranges from 50 to 90% [5, 6]. SCD is especially severe in sub-Saharan Africa [7], with most affected individuals having the most severe form, sickle cell anaemia (SCA). Tragically, about 90% of individuals with SCA (HbSS genotype) in Africa do not live to reach their 18th birthday [2].

Symptoms of SCD typically manifest before a child’s first birthday [8] and can include episodes of pain, swelling in the hands or feet, frequent infections, delayed growth, and anaemia [9]. Over their lifetime, individuals with SCD experience multiple medical and physical complications such as stroke, chronic pain, yellowing of the eyes, priapism, splenic sequestration, acute renal failure, avascular bone necrosis, pulmonary hypertension, heart failure, leg ulcers, and bone necrosis [10].

Living with SCD not only pose physical health challenges to persons living with the condition, but also psychosocial issues [11–13], including stigmatization [14, 15]. Stigma is a distinct attribute, hidden or discernible, that is deeply discrediting [16] and that can severely impact on the quality of life of individuals living with a specific disease [17, 18]. Quality of life (QoL) encompasses an individual’s perception of their position in life in the context of the culture and value systems in which they live, and in relation to their goals, expectations, standards, and concerns [19]. This includes how they feel about their health, material comfort, personal safety, relationships, learning, creative expression, opportunity to help and encourage others, participation in public affairs, socializing, and leisure [20]. Research in different parts of the world suggests that stigma can substantially lower the QoL for individuals living with SCD [21, 22]. Given the above, we conducted an exploratory study in three African countries with the objective of identifying: (1) drivers and facilitators of SCD-related stigma and (2) strategies for mitigating SCD-related stigma.

## Method

This was a qualitative phenomenological study [23] that used focus group discussions (FGDs) and one-on-one in-depth interviews (IDIs) to explore the psychosocial effects of SCD on people living with the condition. The study was carried out in Cameroon, Ghana, and Tanzania, which are countries with a high global SCD burden in Africa [6, 24, 25]. The results presented in this paper are part of the outcome of a larger study on stakeholders’ perspectives on genetic applications for SCD. The comprehensive study methodology and limitations have been previously described [26].

### Study participants

Participants for the study were persons living with SCD and stakeholders with a caregiving role such as parents, family members, healthcare workers, traditional rulers, and religious leaders [27, 28]. All participants were 18 years or older in Ghana and Tanzania, while in Cameroon, they were 21 years and above. This is due to differences in the legal age of adulthood in the respective countries [29]. Potential participants were identified by the lead investigator in each country. Lead investigators were healthcare workers running an SCD clinic or research project and familiar with the study population. Purposive and snowball sampling [27, 28] were used to identify potential participants. Demographics of study participants (Table 1) are presented for Cameron and Ghana only as this information was not consistently collected in Tanzania. Also, final numbers (*n*) may not add-up for some demographic categories as they were not reported by all participants. This limitation has been reported in a methodology paper for the study [26].

**Table 1:** Demographics of study participants

Demographics	Cameroon (*n* = 71)	Ghana (*n* = 89)
Reported gender		
Female	41	57
Male	21	26
Occupation		
Healthcare practitioner	47	59
Student	6	–
Educator	2	7
Religious leader	1	5
Traditional leader	1	3
Civil servant/business owner	9	9
Educational level		
Primary	–	3
Secondary	39	9
Tertiary	32	74
Geographical location		
Rural	21	26
Urban	50	63

### IDIs and FGDs

A semi-structured interview guide was used for conducting the IDIs and FGDs. The guide consisted of questions aimed at exploring personal experiences of SCD-related stigma and social consequences of SCD-related stigma and strategies for mitigating stigma associated with SCD. The interview guides were translated and back translated for interviews conducted in French (Cameroon), Twi (Ghana), and Swahili (Tanzania). The IDIs and FGDs were facilitated by a diverse team of research staff including geneticists; healthcare workers with experience in qualitative and/or SCD research; medical students in residency; and social scientists. The research team met regularly to discuss themes that were emerging from the data [26] and final data were guided by the principle of saturation [30]. In this paper, we report on the results of 36 FGDs and 70 IDIs with different stakeholder groups (Table 2).

**Table 2:** FGD and IDIs at the three sites

Country	FGDs	IDIs	Patients and parents of children with SCD*	Other stakeholder groups
Cameroon	9	18	6	21
Ghana	12	33	14	31
Tanzania	15	19	6	28
Total	36	70	26	80

^*^Total number of FGDs and IDIs

### Data analysis

The IDIs and FGDs were audio-recorded and transcribed verbatim. The transcripts were anonymized and then imported into NVivo, a qualitative data management software, to facilitate analysis [31]. A coding scheme consisting of 4 thematic areas: “social stigma”, “stigma and SCD”, “Stigma other”, and “strategies for reducing stigma”, was applied to the transcripts. These thematic areas were based on the outcome of prior formative research on new-born screening for SCD in Ghana [32]. Transcripts were coded by a researcher (KKK, DB, and JA) from each study site. As was the case, in the data collection phase, the research team met regularly to discuss and review coded transcripts from each of the study site. Differences in applying the coding scheme were discussed with MT and a second researcher and resolved by consensus. Data from the different interviews were triangulated so that data from each component are used to enhance understanding of results generated from the other sets of data.

The coded data were subsequently extracted by NSM for further analyses using three conceptual frameworks: *The Bronfenbrenner’s Ecological Systems Theory* [33], the *health stigma and discriminatory framework* [34], and *the public health framework for reducing stigma* [35]. These different frameworks provide a foundational basis for making meaning on how perceptions around SCD could fuel stigma and discrimination. Bronfenbrenner’s ecological systems theory provides a framework for understanding the determinants of human health, considering the complex interplay of various factors at different ecological levels. These determinants of health encompass not only the person’s immediate settings such as family, school, and workplace but also broader societal and cultural contexts such as personal values, customs, laws, socioeconomic status, and political climate [36]. Bronfenbrenner’s socio-ecological theory has been applied to a similar study on caregiver’s perception of stigma and SCD in Ghana [37]. The *health stigma and discriminatory framework* [36], on the other hand, provides insights into the process of stigmatization as it occurs within the socio-ecological spectrum, while the *public health framework for reducing stigma* [35] speaks to public health strategies and interventions for mitigating disease-related stigma.

Ethics approval for this study was obtained from the participating research institutions. All participants provided written informed consent.

## Results

**Fig. 1:**
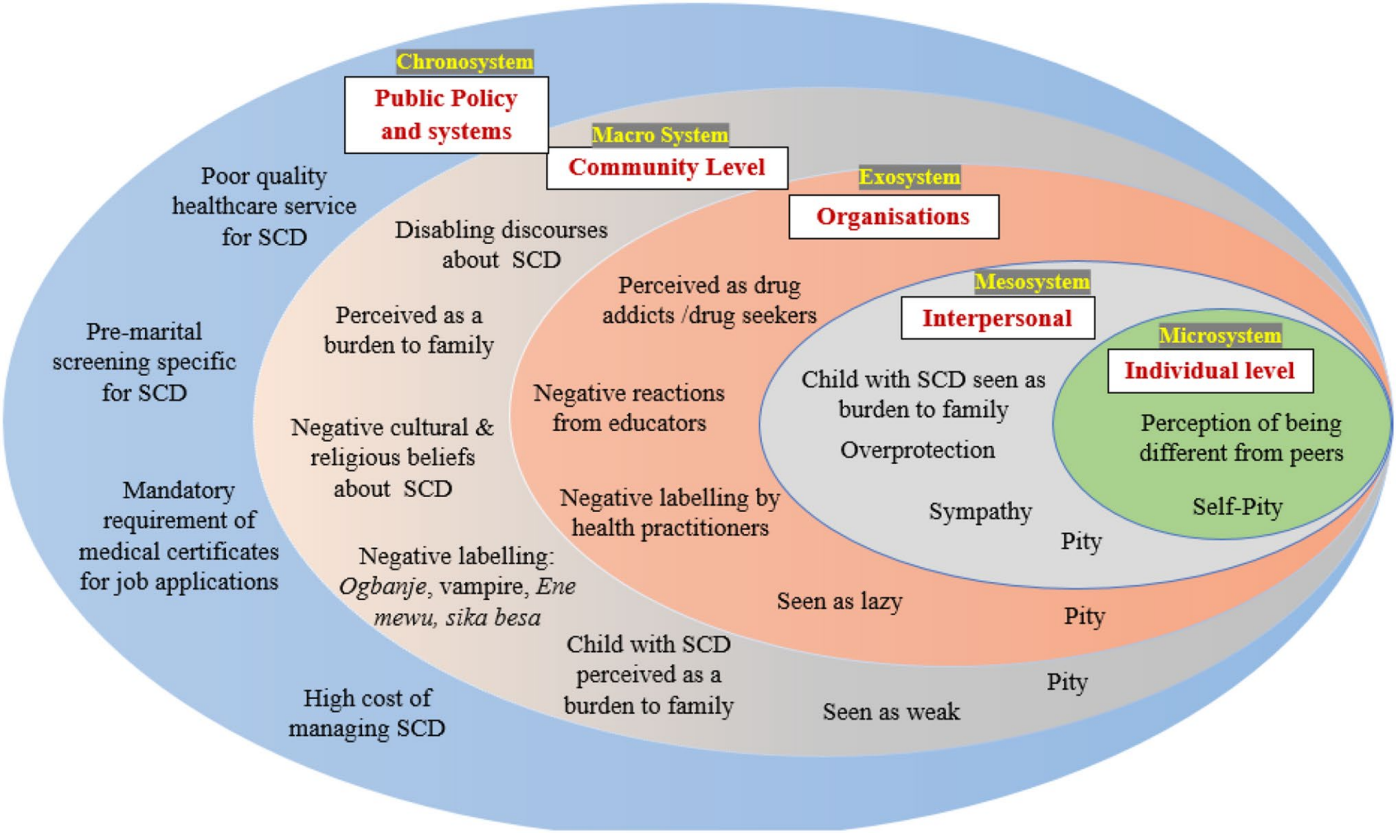
Stigmatizing narratives of SCD at different socio-ecological levels

Analysis of the data through the lens of Bronfenbrenner’s ecological systems theory reveals the pervasive nature of SCD stigma and discrimination across multiple socio-ecological levels (Fig. 1). Pity and explicit bias were common in narratives of how SCD-related stigma can affect the healthcare-seeking behaviours of individuals with the condition, as well as their access to education and employment.

The analysis of the data using the *health stigma and discriminatory framework* allowed us to identify drivers and facilitators of SCD-related stigma and the how it affects individuals living with SCD (Table 3).

**Table 3:** Characterization of SCD stigma based on the health stigma and discriminatory framework

Domains in the health stigma and discriminatory framework	Description of domains	Illustrative examples of SCD-related Stigma
Drivers of SCD-related Stigma	Inherently negative influences that drive health-related stigma	Frequent crises and hospitalizations
		Discrimination based on physical features (e.g. yellow eyes)
		Association of SCD to early mortality
		Social judgement
		Self-blame and denial
		Hereditary nature of SCD leads to stigmatization of families
		Regular need for blood transfusions
		Being called a Sickler
Facilitators of SCD-related stigma	Positive or negative influences that drive health-related stigma	Supernatural beliefs on the causes of SCD (witchcraft)
		Cultural beliefs
		Pity/sympathy from peers and the community
		Viewed as weak
Manifestations of SCD-related stigma	Experiences and practice of stigmatization	Employment discrimination
		Social isolation (friends and peers)
		Internalization of stigma
		Discrediting narratives of SCD
		Discourteous treatment at health facilities, schools, workplace, and community
		Social discrimination in intimate relationships
Outcomes of SCD-related stigma		Explicit bias by healthcare workers negatively impacts the health-seeking behaviour of persons living with SCD
		Status loss: patients feel diminished and valueless
		Challenges integrating people living with SCD into the workplace Othering

### Drivers and facilitators of SCD-related stigma

Common drivers and facilitators of SCD-related stigma included self-blame, prejudice, cultural beliefs surrounding SCD, social norms, and expressions of pity.

I can categorize the stigma in 3 ways; First, people are being told that the life span of a person who has sickle cell is low. Second, because of jaundice, most people associate it with superstitious beliefs. Thirdly, there is a social misconception that the disease can be spread by touching, or associating with, the patient, or sometimes they think the patient may bring misfortune in the family or community and even in marriage. (Ali, IDI, Health Care Provider, Tanzania)

#### Prejudice related to a perceived low life expectancy

Prejudice is characterized by preconceived opinions, attitudes, or judgments formed by individuals or groups based on limited or inaccurate information about a specific person or group. Some participants mentioned that the primary cause of prejudice towards persons living with SCD stems from a widespread belief that persons with SCD are unlikely to live past their 21st birthday.

When they say she, or he, has sickle cell, it is synonymous with death. It’s like she will die soon, he will die soon. I cannot count how many of them have come to me as medical personnel when they were not yet 21 to ask if it is true that they will die before 21 years. (Kameni, FGD, Health Care Provider, Cameroon)

#### Sociocultural beliefs around SCD

Some participants narrated how cultural beliefs that associate SCD with mystical elements have led to children with SCD being labelled as “witches” and wizards, and how this misconception leads to biased attitudes and actions directed at individuals with SCD.

People think that they are witches and wizards especially when they see their ulcers and the protruding belly. People don’t want to come closer to them and their family members abandon them. (Makena, IDI, Health Care Provider, Ghana)

#### Stigma marking and negative labelling of individuals living with SCD

Stigma marking involves affixing derogatory labels or stereotypes to individuals or groups due to specific characteristics or conditions that they possess**.** Across all the study sites, terms such as sorcerers, *ogbanjes, and vampires* were used to describe individuals living with SCD. “Ogbanje” is an Igbo (Nigeria) expression that describes someone who comes and goes [38, 39]. It is also used in Cameroon, to refer to a child believed to be possessed by an evil spirit and that will die young and re-enter their mother’s womb to be born again. There are similar terminologies in Ghanaian [15]

They are called “ene mewu, oshina wo tisae” today I will die, tomorrow will live. (Jojo, FGD, Family of Individual with SCD, Ghana).

This negative labelling may have arisen due to the need for individuals living with SCD to undergo frequent blood transfusions due to anaemia.

They are treated as sorcerers, vampires. They really suffer. I had a case like this where a young girl asked me: doctor, did I steal this disease? Look at how I am marginalized. (Emcee, FGD, Healthcare Provider, Cameroon)

In the above quote, vampire was used as a metaphorical concept to refer to people who consume human blood for sustenance.

#### Stigma marking due to perceived physical deviance

Physical complications associated with SCD, including delayed growth, late sexual maturation, bone age retardation, small body mass, delayed menarche, and hypogonadism, often served as markers that lead to stereotyping and discrimination against persons with SCD. According to some participants, these physical traits were sometimes erroneously attributed to witchcraft or spiritual curses, further highlighting how the convergence of cultural beliefs, negative labelling, and misconceptions could perpetuate SCD-related stigma.

A sickle cell patient, in terms of growth, doesn’t grow as you, the normal person. At school, he is a different person. He cannot play very well with his friends. At any given minute he collapses. (Aya, IDI, Traditional healer, Cameroon)

These community perceptions of physical deviance, when misunderstood by peers, can make children with SCD more vulnerable to being singled out and subjected to teasing and bullying.

I remember back in school, my friend who had sickle cell disease would not be allowed to join us to play football. I am ok with the idea of not playing football but the way they sacked him is the issue. “Go away”, “you are a Sickler”. The way we talk can prevent them from coming out as people living with sickle cell disease. (Abieko, FGD, Family of Individual with SCD, Ghana)

#### Stigma marking due to frequent hospitalizations

Frequent pain crises and hospitalizations are common challenges for individuals with SCD and can unfortunately be a driver of stigmatization. Sometimes, the negative labelling is perpetuated by healthcare workers and therefore a major concern, as healthcare professionals play a crucial role in the care and support of patients.

In Komfo Anokye Hospital, they have nicknamed me ‘Efiewura” meaning landlord. Then through Joe (research assistant), I got to know Dr ABC who operates a private hospital. When I got to the hospital, the nurse started shouting at me saying I come there to worry them and I should hurry and die, since we don’t live for long. They call me all sorts of names such as ‘I will die tomorrow’. (Lumusi, FGD, Individual with SCD, Ghana).

### Manifestations of SCD-related stigma

The study identified four primary manifestations of SCD-related stigma which include social discrimination, implicit bias, pity, and social isolation. These frequently occurred in schools, healthcare facilities, and the workplace.

#### Social discrimination: access to education and lived experiences in schools

Participants in the study recounted instances where children living with SCD and or their parents experienced discrimination in schools. These included schools requesting children with SCD to wear distinguishable clothing items, essentially singling them out and making them feel different or othered.

I was told by one mother that the teachers told the child to attach a red cloth on his shirt, meaning he is a dangerous person, whoever sees him will know that one is dangerous. (Senzo, IDI, Traditional Healer, Tanzania)

Social discrimination with respect to access to education was another prevalent theme that emerged from the study, and there were stories of how some parents held the belief that investing in the education of a child with SCD might not be as valuable compared to a sibling who does not have SCD.

I accompanied a friend to a sickle cell patient’s birthday party, and she was sharing her experience. She said that she had to drop out of school for her younger brother to go to school because her parents saw that she wouldn’t live long, so they shouldn’t waste their money on her. (Fifi, FGD, healthcare provider, Ghana).

#### Social discrimination: dating and marriage

Another common manifestation of social discrimination against adults with SCD was in the context of dating and marriage. Some participants shared how within their communities SCD is stigmatized and perceived as a form of witchcraft. Consequently, many families discourage their relatives from entering into romantic relationships with individuals living with SCD.

Sometimes they see it as witchcraft. They forbid their relative to have a relationship with me because they are afraid of what will be the outcome. (Sango, IDI, Individual with SCD, Cameroon)

#### Social discrimination: inequities in access to employment

The study also identified employment-related social discrimination as a significant concern. This discrimination can manifest in various ways, such as potential employers being reluctant to hire individuals with SCD due to misconceptions about the condition or concerns about frequent sick leave.

When you go and ask for a job, and you mention that I have this [sickle cell] disease they will tell you there are no jobs. If you did not say it, you would get the job. So sometimes you may conceal it to see if you will get a job. But you will be surprised later when you say, I have sickle cell disease they will expel you from the job (Halima, FGD, Individual with SCD, Tanzania)

#### Stigma manifesting as pity

While there was general acknowledgment that individuals with SCD and their families often experience discrimination, it is worth noting that some participants held a contrary view. They expressed that what is characterized as SCD-related stigma is, in many cases, an expression of pity, sympathy, or compassion rather than outright discrimination.

Assuming you are playing football and you are tackling they will say be careful you don’t break his bones, so boys as you are playing with him you don’t have to struggle with him because he is not strong. (Hibo, FGD, Individual with SCD, Ghana)

Persons who held this view also suggested that if there was any form of stigmatization, it was more likely to be internalized stigma. Internalized stigma occurs when individuals with a particular disease or characteristic accept and incorporate negative stereotypes or beliefs about the disease into their self-identity.

I think it is the patient who feels stigmatized. Because the people around him often feel sorry for him, that frustrates him. (Ngwa, FGD, Health Care Provider, Cameroon)

### Outcome of SCD-related stigma

#### Explicit bias by healthcare workers

In some instances, negative narratives of SCD filtered into the healthcare system, and it was not uncommon for healthcare professionals to inadvertently propagate SCD-related stigma and discrimination.

The nurses are the worse culprit in terms of stigma saying all sorts of stigmatizing words such as *okyenena mewu*- I will die tomorrow, *sika besa*- money will finish. (Della, FGD, Individual with SCD, Ghana)

#### Social isolation due to stigma marking

The study findings reveal that stigma marking was common in educational establishments, particularly in primary and secondary schools, resulting in the segregation of students with SCD by their peers.

So, when it is announced in class that you should not beat this student because of the disease, then fellow students will start running away from him. That’s where stigma begins. (Nuru, IDI, Traditional Healer, Tanzania)

Experiences of negative social interactions and feelings of being “othered” may cause individuals with SCD to withdraw and isolate themselves from social interactions and activities.

You don’t grow big, and your size declines all the time, and you are laughed at. When the child is laughed at, he does not want to go to school. (Poku, FGD, Family of an individual with SCD, Ghana)

### Proposed strategies for mitigating SCD-related stigma at different socio-ecological levels

To identify strategies for mitigating SCD-related stigma, we applied the *public health framework for reducing stigma* to our dataset. Four key strategies emerged, namely increased SCD advocacy, stakeholder education, counselling for patients, and improved clinical management of SCD (Table 2).

#### Strategy 1: Increase SCD advocacy and awareness campaigns at policy and community level

Participants emphasized the critical need for public awareness and education about SCD.

When people are not knowledgeable, when they are not sensitized about certain issues, that is when they stigmatize the patients. The approach will be to educate, to sensitize the population, make them know that whatever the situation, sickle cell is a normality and not a fatality. (Tchami, IDI, Administrator, Cameroon)

#### Strategy 2: Continuous education of healthcare workers and community leaders

It was recommended that SCD education should specifically target healthcare workers and community leaders as the attitudes and behaviours of these stakeholder groups can profoundly influence how individuals with SCD are viewed by their communities.

Education should target different groups, including healthcare workers and leaders that are well respected in communities, such as religious heads. The reason is that people listen to us. (Thema, IDI, Religious leader, Ghana)

It was suggested that mainstream media and lessons on SCD in school curricula could help correct misconceptions about SCD.

You can have health classes in schools or have television programs that broadcast about sickle cell disease and stigma, that way you can reach many people at the same time. Or you can even use the radio to reach those who don’t have televisions. (Kwate, IDI, Health Care Provider, Tanzania)

#### Strategy 3: counselling of persons living with SCD.

The second recommended strategy was providing counselling to individuals with SCD on the impact of the condition on their psychosocial well-being, and strategies for addressing stigma and discrimination.

I think the patients themselves should be made to believe that they are not any different from other people, just like (the other group member) said. Some feel inferior already, which aggravates the degree of stigmatization. (Kwame, FGD, Health Care Provider, Ghana)

#### Strategy 4: improved clinical management of SCD

For some participants, clinical interventions aimed at improving health outcomes, and by extension QoL of individuals with SCD could go a long way to reduce stigmatization of the disease.

The stigmatization comes as a result of the outcome of the disease; you are going to die. I think if there is proper management of SCD, it will help reduce stigma. (Osei, FGD, Health Care Provider, Ghana)

## Discussion

Using three frameworks for stigma in  health and disease, we have provided a comprehensive understanding of how SCD-related stigma operates at various socio-ecological levels (Fig. 1), and its impact on the QoL of persons living with SCD. The study also highlights the drivers, facilitators, manifestations, and outcome of SCD (Table 3), and strategies for mitigating SCD-related stigma (Table 4). The results demonstrate that discrediting narratives of SCD contribute profoundly to status loss, low self-esteem, social isolation, depression, and discrimination in access to essential services such as healthcare, employment, and education.

**Table 4:** Proposed strategies for mitigating SCD-related stigma based on the public health framework for reducing stigma

Intervention Strategy/mechanism based on the framework	Mechanisms for mitigating discrimination	Mechanisms for mitigating psychological impact of stigma
Advocacy	Condemn explicit bias by health care workers	Speak out against negative stereotyping of persons living with SCD, e.g. in the media, churches, community meetings
	Condemn workplace practices that discriminate against persons living with SCD	
	Encourage employers to make accommodations for persons living with SCD to flourish in the workplace	
Contact	Facilitate SCD awareness programmes in communities where SCD is stigmatized	Counselling for adults living with SCD
		Encourage persons with SCD and their caregivers to join SCD support group
Education	Provide training to healthcare workers, educators, and communities on the harms of stigma and negative labelling	Educate people living with SCD about the impact of self-stigma
	Leverage on mainstream media such as radio newspapers and television to raise public awareness of SCD	Teach individuals with SCD skills for building self-esteem and coping with discriminatory treatment
	Incorporate courses or modules on SCD into school curricula	Educate teachers on processes that will accommodate the needs of students with SCD while allowing them to effectively participate in school activities
Regulation	Develop and promote policies and programmes that address discriminatory practices against persons living with SCD	Advocate for governments to prioritize SCD as part of public health programmes and to promote public education on SCD

A very concerning finding of this study is explicit bias, negative labelling, and stigmatization from healthcare practitioners and educators. Similar studies in Ghana, the Middle East, and South America have reported that explicit bias by healthcare workers entrenches public stereotyping and social marginalization of individuals living with SCD [40–43] and may inevitably see healthcare providers delivering inferior health care services to individuals with SCD [44].

Addressing SCD-related stigma and discrimination would require a multistakeholder approach with a focus on education and raising public awareness about SCD and dispelling myths and misconceptions about SCD. For the public, this may be via mainstream media for example, public health service announcements, documentaries/movies, and storytelling by individuals living with SCD, and should ideally emphasize the reality that individuals with SCD are not defined solely by their health condition. At the level of schools, incorporating SCD topics into health education and life sciences classes may help improve public education about SCD at an early stage. Education of healthcare workers should cover the psychosocial burden individuals with SCD face, strategies for providing unbiased and equitable care, and the detrimental effects of negative labelling on health-seeking behaviour. Irrespective of the stakeholder group, educational programmes should emphasize that stigmatization and social discrimination of persons living with a disease represent a social injustice.

### Study limitations

A major limitation of this study is that during the FGDs, some individuals with SCD and their family members became emotional when discussing feelings of stigmatization. This may have influenced how other participants in the same FGDs responded to the questions. However, the IDIs allowed for more one-on-one discussions, whereby participants could freely express their views without concerns of how it may affect others. A second limitation of this study is that the interpretation of the findings was not validated with the study participants, and therefore, we may have introduced some researcher bias and preconceptions in our interpretation of the study results.

## Conclusion

Based on our experience working in SCD programmes across Africa [45–47], we are of the view that the effectiveness of any stigma mitigating intervention would require a patient-centred and community-driven approach. This will involve empowering SCD patient support groups and community leaders to lead advocacy and educational campaigns; investing in SCD specific training for healthcare workers; and lobbying African governments to prioritize SCD programme, allocate resources for SCD education, and establish guidelines and protocols that promote non-discriminatory practices within healthcare systems.

## Data Availability

The dataset for this article is available upon reasonable request from the corresponding author.
